# APETx4, a Novel Sea Anemone Toxin and a Modulator of the Cancer-Relevant Potassium Channel K_V_10.1

**DOI:** 10.3390/md15090287

**Published:** 2017-09-13

**Authors:** Lien Moreels, Steve Peigneur, Diogo T. Galan, Edwin De Pauw, Lászlo Béress, Etienne Waelkens, Luis A. Pardo, Loïc Quinton, Jan Tytgat

**Affiliations:** 1 Toxicology and Pharmacology, KU Leuven, Leuven 3000, Belgium; lien.moreels@kuleuven.be (L.M.); steve.peigneur@kuleuven.be (S.P.); diogo.teodorogalan@kuleuven.be (D.T.G.); 2 Laboratory of Mass Spectrometry—MolSys, University of Liege, Liege 4000, Belgium; e.depauw@ulg.ac.be (E.D.P.); loic.quinton@ulg.ac.be (L.Q.); 3 Immunology and Rheumatology, Section of Peptide Chemistry, Hannover Medical School (MHH), Hannover 30625, Germany; dr.beress@t-online.de; 4 Laboratory of Protein Phosphorylation and Proteomics, KU Leuven, Leuven 3000, Belgium; etienne.waelkens@kuleuven.be; 5 Oncophysiology Group, Max Planck Institute for Experimental Medicine; Göttingen 37075, Germany; pardo@em.mpg.de

**Keywords:** sea anemone peptide, APETx, potassium channel, K_V_10.1, cancer

## Abstract

The human ether-à-go-go channel (hEag1 or K_V_10.1) is a cancer-relevant voltage-gated potassium channel that is overexpressed in a majority of human tumors. Peptides that are able to selectively inhibit this channel can be lead compounds in the search for new anticancer drugs. Here, we report the activity-guided purification and electrophysiological characterization of a novel K_V_10.1 inhibitor from the sea anemone *Anthopleura elegantissima*. Purified sea anemone fractions were screened for inhibitory activity on K_V_10.1 by measuring whole-cell currents as expressed in *Xenopus laevis* oocytes using the two-microelectrode voltage clamp technique. Fractions that showed activity on Kv10.1 were further purified by RP-HPLC. The amino acid sequence of the peptide was determined by a combination of MALDI- LIFT-TOF/TOF MS/MS and CID-ESI-FT-ICR MS/MS and showed a high similarity with APETx1 and APETx3 and was therefore named APETx4. Subsequently, the peptide was electrophysiologically characterized on K_V_10.1. The selectivity of the toxin was investigated on an array of voltage-gated ion channels, including the cardiac human ether-à-go-go-related gene potassium channel (hERG or Kv11.1). The toxin inhibits K_V_10.1 with an IC_50_ value of 1.1 μM. In the presence of a similar toxin concentration, a shift of the activation curve towards more positive potentials was observed. Similar to the effect of the gating modifier toxin APETx1 on hERG, the inhibition of Kv10.1 by the isolated toxin is reduced at more positive voltages and the peptide seems to keep the channel in a closed state. Although the peptide also induces inhibitory effects on other K_V_ and Na_V_ channels, it exhibits no significant effect on hERG. Moreover, APETx4 induces a concentration-dependent cytotoxic and proapoptotic effect in various cancerous and noncancerous cell lines. This newly identified K_V_10.1 inhibitor can be used as a tool to further characterize the oncogenic channel K_V_10.1 or as a scaffold for the design and synthesis of more potent and safer anticancer drugs.

## 1. Introduction

Cancer is still one of the leading causes of death worldwide. In 2012 about 14.1 million new cancer cases and 8.2 million cancer deaths were reported [1]. The WHO reports that in 2015, the number of cancer deaths has risen to 8.8 million. This corresponds to nearly 1 in 6 deaths [2]. Since the population continues to grow and age, these numbers are expected to increase. It is predicted that there will be 21.7 million new cancer diagnoses and 13 million cancer deaths in 2030. These numbers are probably even an underestimation since the increased cancer risk in developing countries due to poor diet, smoking etc. was not taken into account [1,3]. It is clear that the burden of cancer should be reduced. Therefore, there is an urgent need for more effective and safer anticancer drugs.

The voltage-gated potassium channel human ether-à-go-go 1 (hEag1, K_V_10.1) represents an interesting cancer target because it is overexpressed in a wide range of human tumor cells and tissues [4,5,6,7,8,9,10] but is virtually undetectable in healthy tissue outside the central nervous system (CNS) [6,11,12,13]. Overexpression of rat Eag1 in NIH-3T3 cells induced features that are characteristic for malignant cell transformation [14]. Since then, several approaches inhibiting either channel expression or activity by siRNA [15], small molecules [16,17,18] and monoclonal antibodies [19,20] has led to inhibition of proliferation of tumor cells both in vitro and in vivo. Unfortunately, in vivo efficacy and selectivity remains a major issue for most of these compounds. Many small compounds, such as astemizole and imipramine, also block the cardiac human Eag-related gene (hERG, K_V_11.1) and can therefore be arrhythmogenic [21,22].

Although several venom peptides are known to exhibit therapeutic effects by interacting with ion channels [23], only one venom peptide, κ-hefutoxin 1, has been described in literature to show an inhibitory effect on the K_V_10.1 channel [24]. This peptide was originally isolated from the venom of the Asian forest black scorpion *Heterometrus fulvipes* by Srinivasan and colleagues [25].

In order to identify novel K_V_10.1 inhibitors, we electrophysiologically screened fractions of the sea anemone *Anthopleura elegantissima.* The marine environment is considered to be an underexploited but very interesting source of novel anticancer drugs [26,27,28,29,30,31]. Moreover, compounds extracted from venomous marine invertebrates, such as sea anemones, contain a wide array of bioactive compounds [32,33]. An interesting example is the ShK peptide from the sea anemone *Stichodactyla helianthus*, which is able to block both K_V_1.1 and K_V_1.3 in the low picomolar range. An analogue of this peptide, named Dalazatide (Shk-186)) with improved selectivity for K_V_1.3 was developed [34] and has currently completed Phase I clinical trials for the treatment of autoimmune diseases [35,36].

In this work, we present the identification of a novel sea anemone peptide APETx4. The effect of this peptide on K_V_10.1 and other ion channels was electrophysiologically investigated. The antiproliferative, cytotoxic and pro-apoptotic activity of APETx1 was evaluated on various cancerous and non-cancerous cell lines.

## 2. Results

### 2.1. Activity-Guided Purification and Identification of A Novel K_V_10.1 Inhibitor

To identify novel inhibitors of the potassium channel K_V_10.1, previously purified fractions from the sea anemone *A. elegantissima* [37] were screened for inhibitory activity on K_V_10.1. The fraction showing inhibitory effect was further purified by RP-HPLC. The monoisotopic molecular mass of the purified peptide was determined by MALDI-TOF MS (4651.02 Da) and ESI-FT-ICR MS (4650.9974 Da) (Figure S1).

**Figure 1:**
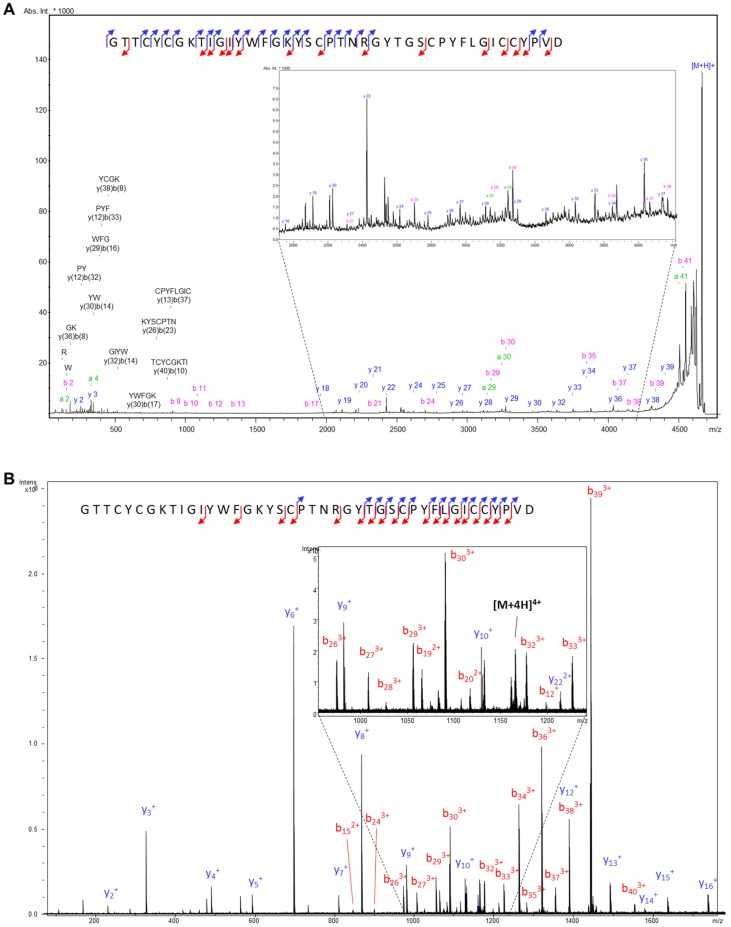
MS/MS spectra characterizing the APETx4 sequence. (**A**) MALDI-PSD-TOF/TOF of the [M+H]^+^ species @*m/z* 4651.02 and (**B**) ESI-FT-ICR MS/MS (CID) of the [M+4H]^4+^ species @*m/z* 1163.76. The combination of the two spectra led to the characterization of the whole sequence (34 peptide bonds broken over 38). The isobaric amino acids L and I have been attributed by sequence homology with APETx1 and APETx3.

Reduction of the peptide by TCEP yielded a monoisotopic molecular mass of 4657.0334 Da using ESI-FT-ICR MS. This data indicates that 3 disulfide bonds are present in the purified, oxidized peptide (Figure S2). This molecular mass does not correspond to any known compounds derived from *A. elegantissima*. The sequence of this novel peptide was determined by a combination of Post-Source Decay analysis (MALDI-LIFT-TOF/TOF MS/MS) and Collision-Induced Dissociation (CID-ESI-FT-ICR MS/MS). The determined amino acid sequence and MS/MS spectra are shown in Figure 1.

**Figure 2:**
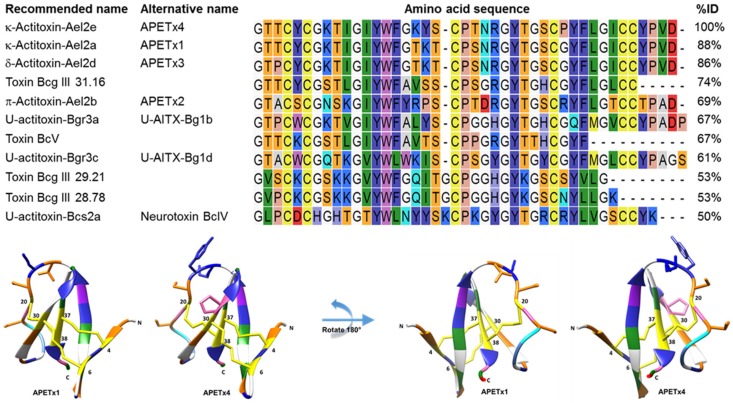
In the upper panel, a multiple sequence alignment of APETx4 and its homologous sea anemone peptides is shown. Peptide names recommended by UniProt and alternative names are given. A Clustal Omega sequence alignment was performed using CLC Main Workbench. Amino acid residues are colored according to the RasMol amino color scheme. Percentages of identity (% ID) were obtained using standard protein BLAST. In the lower panel, the amino acid sequence of APETx4 was modeled on an averaged structured obtained from the solution NMR structure of APETx1 (PDB ID: 1WQK) using Modeller and Chimera. The amino acid residues are colored according to the RasMol amino color scheme. The 5 amino acid residues that differ between APETx1 and APETx4 are displayed as sticks. The C- and N-terminal residues and the cysteine residues (4, 6, 20, 30, 37 and 38) are indicated.

A standard protein Basic Local Alignment Search (BLAST, NCBI) using the blastp algorithm was performed and 26 Blast hits were identified. Only 11 of these hits were found in the UniprotKB Protein knowledgebase to exist with experimental evidence at the protein level. All of them originate from the Actiniidae family, which is the largest and most diverse family of the Actiniaria order (sea anemones) [38]. A multiple sequence alignment of the purified peptide with these 11 homologues sea anemone peptides is shown in Figure 2.

The novel peptide shows an 88% and an 86% identity to respectively APETx1 and APETx3 and was therefore named APETx4 (Figure 2). If the nomenclature suggested by King and colleagues [39,40] is followed, the peptide can be named κ-actitoxin-Ael2e or in short κ-AITX-Ael2e. The amino acid sequence of APETx4 was deposited in the UniProt Knowledgebase (UniProtKB) under accession number C0HL40.

Due to the high sequence similarity between APETx4 and APETx1, the solution NMR structure of APETx1 (PDB ID: 1WQK) was used as a template for homology modeling of APETx4. First, an average structure of the 25 final solution structures of APETx1 was generated in Chimera. Secondly, a homology model of the target sequence of APETx4 from the template APETx1 was made using Modeller and Chimera (Figure 2 and Figure S3).

### 2.2. APETx4 is A Gating Modifier of K_V_10.1

**Figure 3:**
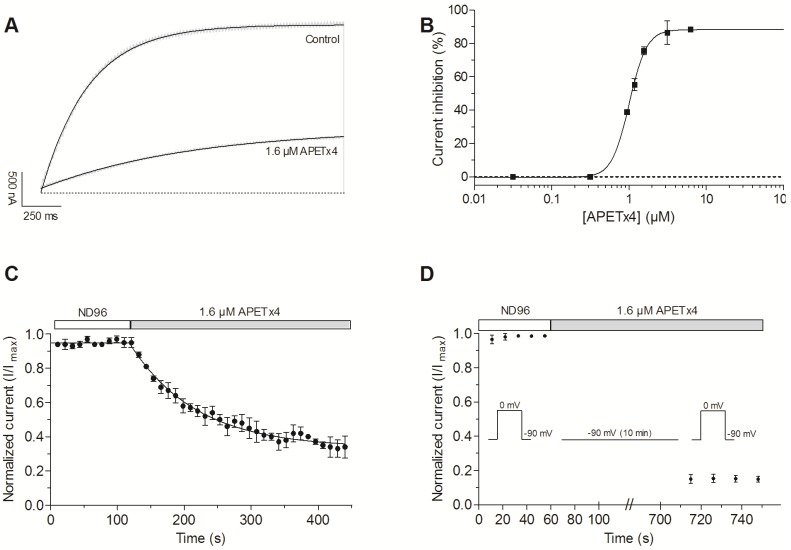
Concentration- and state-dependent effect of APETx4 on evoked K_V_10.1 currents. (**A**) Representative K_V_10.1 current evoked by a 2 s pulse to 0 mV from the holding potential in control and toxin condition are shown in light gray. An exponential one phase association equation was used to fit the evoked currents (black lines); (**B**) Concentration-dependency plot fitted with a logistic equation shows the current inhibition (%) in function of APETx4 concentration; (**C**) The normalized current (I/I_max_) was plotted versus the APETx4 incubation time. The time points were fitted with a one-phase decay exponential equation; (**D**) The state-dependency plot shows the normalized current in control conditions and in toxin condition. During the APETx4 addition the cell membrane potential was clamped at −90 mV for 10 min. All measurements were performed in external ND96 solution.

Once the active peptide was purified and identified, the effect of APETx4 on K_V_10.1 was further electrophysiologically characterized. After addition of 1.6 µM APETx4, a 76 ± 2% inhibition of the outward current was observed. To investigate the effect of APETx4 on the rate of activation, the evoked K_V_10.1 currents were fitted with the exponential equation *y* = *y*_0_ + y_max_ (1 – e^−*t*/τ^), where *y*_0_ is the evoked current at *t* = 0 s, *y*_max_ is the maximum current, and τ is the time constant. APETx4 appears to reduce the rate of K_V_10.1 activation. In control conditions, the time constant τ was 376 ± 60 ms. After toxin addition (1.6 µM), τ increased to 1209 ± 229 ms (*n* = 3, *p* < 0.05) (Figure 3A). The rise time *t*_r_ (ms) was also calculated from the raw data and refers to the time the current rises from 10% to 90% of its final value. APETx4 increased the rise time from 653 ± 42 ms in control conditions to 1246 ± 96 ms (*n* = 3, *p* < 0.05).

A concentration-response curve was generated by measuring the current inhibition (%) as a function of increasing concentrations. The data points were fitted with a logistic sigmoid function, the calculated IC_50-_value was 1.01 ± 0.01 µM, the Hill coefficient was 3.8 ± 0.3 and the maximal current inhibition was 88.3 ± 0.9% (Figure 3B). Figure 3C shows the observed normalized current (I/I_max_) versus the incubation time with APETx4. The time points were fitted with the exponential one phase decay equation *y* = *y*_∞_ + (*y*_0_ − *y*_∞_) e^−*t*/τ^, where *y*_∞_ represents the normalized current that is reached at infinite times (plateau) and *y*_0_ is the normalized current before toxin addition. After addition of 1.6 µM APETx4, the observed normalized current gradually decreased with a time constant of τ = 97 ± 7 s.

The state-dependency of the inhibition was studied by holding the cell potential at −90 mV for 10 min during the addition of 1.6 µM APETx4 to the oocyte. After keeping the channels in a closed state at the holding potential, the normal pulse protocol to 0 mV was resumed. The current upon the first stimulation was reduced by 85.2 ± 1.9% in comparison with the control current measured before toxin addition. This seems to indicate that the toxin is able to bind the channel in its closed state (Figure 3D).

**Figure 4:**
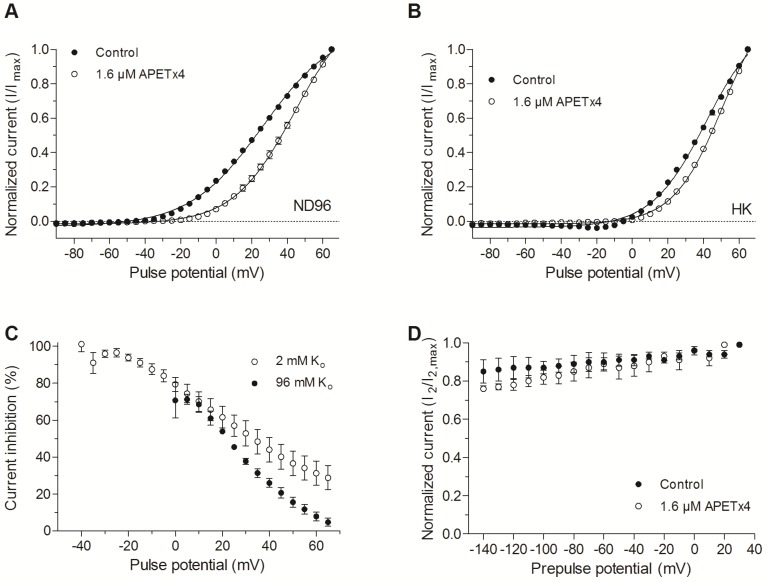
Voltage-dependent effect of APETx4 on evoked K_V_10.1 currents. (**A**) Normalized currents elicited in ND96 solution (2 mM K_o_) were plotted versus the applied pulse potentials (mV) in control (●) and toxin condition (○). The data points were fitted with the Boltzmann equation; (**B**) Normalized currents elicited in HK solution (96 mM K_o_) were plotted versus the applied pulse potentials (mV) in control (●) and toxin condition (○). The data points were fitted with the Boltzmann equation; (**C**) The inhibited current (%) observed after 1.6 µM APETx4 addition in ND96 (○) and HK (●) solution was plotted versus the applied pulse potentials; (**D**) Effect of APETx4 on the inactivation properties of K_V_10.1. The non-inactivating channel fraction (I_2_/I_2,max_) was plotted against the corresponding prepulse potential (mV) in control (●) and toxin condition (○).

APETx4 modifies the voltage dependence of K_V_10.1. In the presence of 1.6 μM APETx4, a 17.7 ± 1.1 mV shift of the activation curve towards more positive potentials was observed (*n* = 3; *p* < 0.05). The half-maximal voltage (*V*_1/2_) shifted from 26.4 ± 0.9 mV in control condition to 44.1 ± 0.7 mV after addition of 1.6 µM APETx4. The slope factor k shifted from 20.7 ± 0.6 mV in control condition to 17.2 ± 0.3 mV in toxin condition (*n* = 3, *p* < 0.05) (Figure 4A).

In a high potassium solution (HK solution, 96 mM K_o_), V_1/2_ shifted from 41.3 ± 1.4 mV in control condition to 50.9 ± 0.8 mV after addition of 1.6 µM APETx4, a shift of 9.6 ± 1.7 mV (*n* = 3; *p* < 0.05). The slope factor shifted from 15.3 ± 1.4 in control condition to 13.9 ± 0.8 in toxin condition (*n* = 3, NS). After toxin addition, no inward current was observed at negative pulse potentials (Figure 4B).

To investigate whether the percentage of current inhibition is also voltage-dependent, the non-normalized inhibited current (1-I_t_/I_c_) was plotted against the applied activating potentials. The current inhibition of K_V_10.1 by 1.6 μM APETx4 was reduced at more positive potentials. When a depolarizing pulse from −90 mV to 0 mV was applied, an inhibition of 79 ± 4% could be observed. When a more depolarizing pulse was applied, for example to 65 mV from the holding potential, the K_V_10.1 current was only reduced by 29 ± 6%. In a high potassium solution, a similar effect was observed (Figure 4C).

In order to investigate the effect of APETx4 on the inactivation properties of K_V_10.1, a two-pulse protocol was used as described in [17]. This protocol consists of a variable 1.5 s prepulse step, ranging from −140 mV to 30 mV in 10 mV steps, followed by a 0.5 s test pulse to 30 mV. Figure 4D shows the non-inactivating channel fraction (I_2_/I_2,max_) plotted against the corresponding prepulse potential. I_2_ is the peak current measured during the 30 mV test pulse, I_2,max_ is the maximal peak current elicited by the test pulse. No apparent inactivation was observed in control or toxin condition.

### 2.3. Effect of APETx4 on Different Voltage-Gated Ion Channels

**Figure 5:**
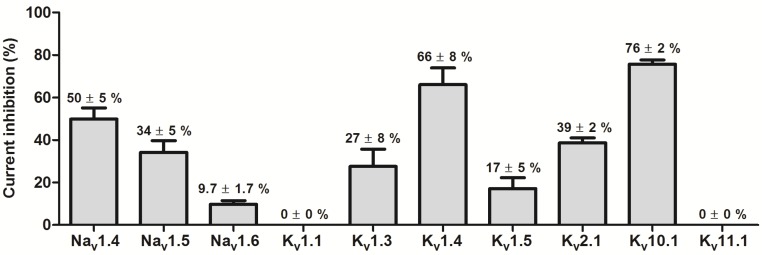
Selectivity screening on a panel of Na_V_ and K_V_ channels. The current inhibition (%) observed after addition of 1.6 µM APETx4 to various channels is displayed in a bar graph. Values are shown as average ± SEM of at least 3 independent experiments.

APETx-like toxins are known to be promiscuous [41], therefore APETx4 was screened against a panel of Na_V_ and K_V_ channels. At a concentration of 1.6 µM, APETx4 exhibited the highest activity on K_V_10.1 (*n* = 3). No significant inhibition was observed for K_V_1.1 (*n* = 11) and K_V_11.1 (*n* = 3). Inhibitions of 50% or less were observed for Na_V_1.4 (*n* = 5), Na_V_1.5 (*n* = 5), Na_V_1.6 (*n* = 3), K_V_1.3 (*n* = 6), K_V_1.5 (*n* = 3) and K_V_2.1 (*n* = 4). A higher percentage of inhibition was observed for K_V_1.4 (*n* = 3) (Figure 5). Figure S4 shows representative traces in control conditions and after addition of 1.6 µM APETx4 of all the screened ion channels. APETx4 appears to be not very selective and also inhibits several other Na_V_ and K_V_ channels.

Since most inhibitors of K_V_10.1 also inhibit the cardiac hERG channel, the effect of APETx4 on hERG was further investigated.

After addition of 1.6 µM or even 10 µM (10 × IC_50_) APETx4, no significant inhibition of the hERGtail current was observed. Since the inhibition of K_V_10.1 by APETx4 and of hERG by APETx1 is voltage-dependent, the voltage-dependent effect of 10 µM APETx4 on hERG was also investigated.

**Figure 6:**
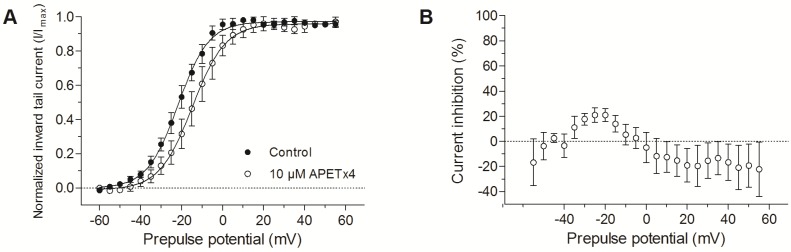
Voltage-dependent effect of APETx4 on hERG inward tail currents. (**A**) Normalized inward tail currents were plotted versus the applied pulse potentials (mV) in control (●) and toxin condition (○). The data points were fitted with the Boltzmann equation. (**B**) The inhibited current (%) observed after 10 µM APETx4 addition was plotted versus the applied pulse potentials.

Normalized current-voltage relationship of the inward tail currents were fitted with the Boltzmann equation. A 7.7 ± 0.4 mV (*n* = 3, *p* < 0.05) shift of the activation curve towards more positive potentials was observed after the addition of 10 µM APETx4 (Figure 6A). The highest inhibition of the tail current (21 ± 5%) was obtained after an activating step to −20 mV. The tail current increased during activation steps to positive potentials (≥0 mV) after APETx4 addition (Figure 6B).

### 2.4. Effect of APETx4 on Cancerous and Non-Cancerous Cell Lines

To investigate whether the observed electrophysiological effects on K_V_10.1-expressing oocytes can be translated into anticarcinogenic in vitro effects, several high-throughput live-cell imaging assays were performed. Proliferation, cytotoxicity and apoptosis assays were performed on 5 different cell lines using the IncuCyte ZOOM system. Table 1 gives an overview of the used cell lines and their presumed K_V_10.1 expression level.

The cell proliferation was quantified in terms of cell confluency (%) over time; data points were collected every hour. APETx4 induced a concentration-dependent antiproliferative effect in all the cell lines except in the healthy fibroblast cell line NIH-3T3. Since some of the affected cell lines do not express K_V_10.1, f.e. LNCaP, it is presumed that the observed effect cannot only be attributed to K_V_10.1 inhibition (Figure S5).

Cytotoxicity was visualized and quantified by addition of the CellTox Green Dye over time. This asymmetric cyanine dye can enter the nonviable cell once the membrane integrity is compromised. This dye is unable to enter viable cells but binds DNA in (or released from) dead cells, thereby enhancing its green fluorescence. All cell lines, except NIH-3T3 became nonviable after addition of APETx4. Interesting to note is that the cytotoxic effect of high APETx4 concentrations on LNCaP and hTERT RPE-1 appeared very rapidly (Figure S5).

**Figure 7:**
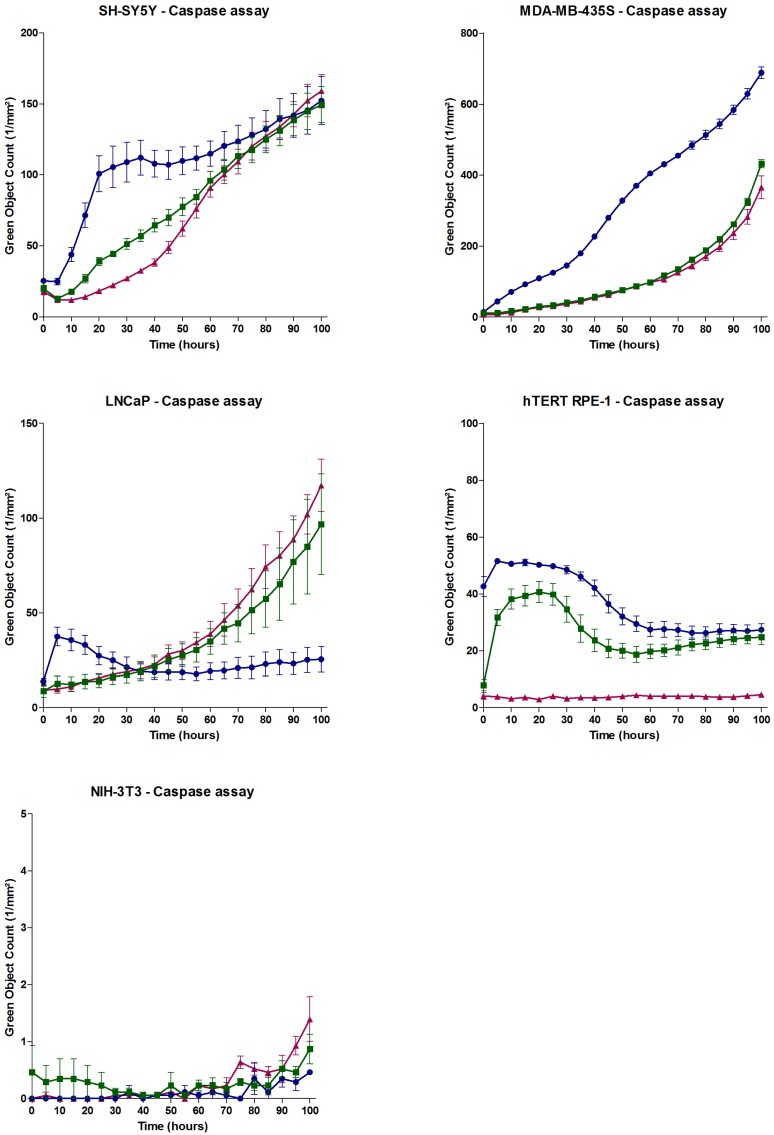
Investigation of the proapoptotic effect of APETx4 on various cell lines using a Caspase-3/7 Reagent. The amount of cells in early apoptosis was plotted as Green Object Count (1/mm²) versus time (hours). The data points represented as red triangles were obtained from apoptosis experiments with cells incubated without APETx4, the green squares represent experiments with 20 µM APETx4 and the blue circles were obtained from experiments with 50 µM APETx4.

The proapoptotic activity of APETx4 was visualized and quantified by the addition of the IncuCyte Caspase-3/7 Reagent. This reagent is an inert and non-fluorescent substrate, which is cleaved by activated caspase-3/7 during early apoptosis. The cleavage of the reagent releases a DNA-binding green fluorescent dye. Similar to the effects observed during the antiproliferative and cytotoxic assays, proapoptotic effects were observed for all the cell lines except for NIH-3T3. In Figure 7 an overview is given for the caspase assays of all the tested cell lines. The green object count (1/mm²) is plotted against the incubation time: A gradual increase in green fluorescent cells was observed for the K_V_10.1-expressing cancerous cell lines SH-SY5Y and MDA-MB-435S. A very rapid response (<60 min) was observed for the cancerous cell line (no K_V_10.1 expression) LNCaP and the K_V_10.1-expressing noncancerous cell line hTERT RPE-1.

Figure S6 provides images taken by the IncuCyte Zoom System for each cell line at 0 h, 24 h and 48 h after changing the growth medium by medium containing 0 µM or 50 µM APETx4. These images show that immediately after toxin addition, the LNCAP and hTERT RPE-1 cells became less viable. For SH-SY5Y and MDA-MB-435S these immediate effects were not clearly observed. Similar images were obtained for the fibroblast cell line NIH-3T3 in control and toxin (50 µM APETx4) condition.

## 3. Discussion

### 3.1. APETx4 is a Gating Modifier that Presumably Binds to the Voltage Sensor Paddle of K_V_10.1

Here, we identified a novel sea anemone toxin (APETx4) that inhibits the K_V_10.1 currents in a concentration- and voltage-dependent manner by binding to the channel in its closed state. We propose that the mode of action of APETx4 on K_V_10.1 is similar to that of APETx1 on hERG [42]. Both toxins shift the activation curve towards more positive potentials. This results in a lower current inhibition (%) at more depolarized potentials, when more channels are open. Our data indeed suggest that APETx4 binds to closed K_V_10.1 channels and reduces the activation rate. More depolarized pulse potentials are necessary to evoke K_V_10.1 currents in the presence of APETx4. A high external potassium concentration (HK) results in small inward currents at a membrane potential around −20 mV in control condition. However, in the presence of APETx4, no inward currents were observed. This suggests that at these negative potentials, the channel is kept in a closed conformation by APETx4. We hypothesize that like APETx1, APETx4 is a gating modifier that presumably binds to the S3b-E2-S4 region (voltage sensor paddle) of K_V_10.1. The high Hill coefficient (~4) suggests possible co-operativity. Co-operativity was also suggested for the interaction between the gating modifier saxitoxin (STX) and hERG [43]. STX also slows down the channel opening and stabilizes the closed state of hERG channels. However, more in depth whole-cell patch clamp experiments as described in [43] are necessary to confirm our hypothesis of cooperative binding.

**Figure 8:**
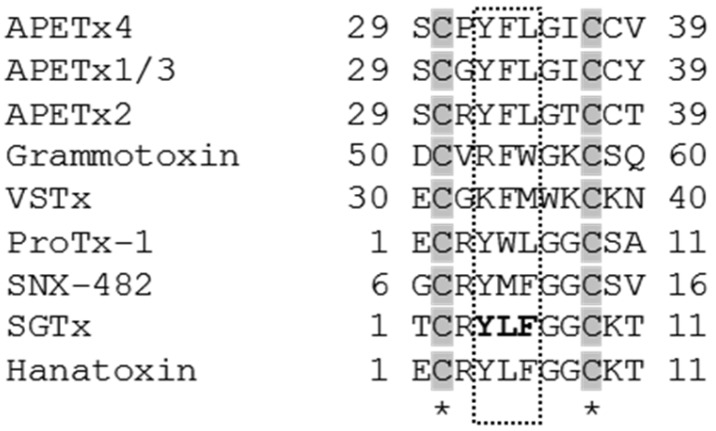
A multiple sequence alignment of various gating modifier toxins was performed using Clustal Omega (EMBL-EBI). Cysteine residues are highlighted in grey and the hydrophobic residues; Y4, L5, F6 that are functionally important for the activity of SGTx on the K_V_2.1 channel.

APETx1 is proposed to bind to the voltage sensor paddle of hERG. In particular, the negative charge of E518 is expected to form an electrostatic interaction with the toxin [44]. Several other kinds of gating modifier toxins also bind to the extracellular exposed part of the S3–S4 domain. More precisely by binding to a glutamic acid residue and several hydrophobic amino acid residues [45]. An example of these toxins is Hanatoxin (HaTx1) from the tarantula *Grammostola spatulata*. This toxin modifies the channel gating of K_V_2.1 by binding to two hydrophobic (I273 and F274) residues and to a glutamic acid residue (E277) [46]. Takahashi et al., suggest that the combination of a hydrophobic patch and charged amino acid residues on the toxin surface is responsible for the binding of gating modifiers to ion channels [47]. Figure S3 shows that APETx4 contains such a hydrophobic patch and charged residues on one side of the toxin. A sequence alignment of several known gating modifiers (Figure 8), shows that many of them, including APETx4 consist of a hydrophobic triad. Wang and colleagues showed that the active surface of the gating modifier SGTx is composed of a patch of important hydrophobic residues; Y4, L5, F6 and W30 [48]. The hydrophobic residues Y32, F33 and L34 of APETx4 correspond to Y4, L5 and F6 of SGTx.

### 3.2. APETx4 is able to Distinguish the Oncogenic K_V_10.1 Channel from the Cardiac hERG Channel

The drawback of many K_V_10.1 inhibitors is that they inhibit hERG with a similar or higher affinity. Inhibition of this cardiac channel can cause long QT syndrome, which can lead to arrhythmia and sudden death [49]. However, hERG is also overexpressed in several cancer cells and human tumors and is therefore also an anticancer target [50,51,52]. Since hERG liability is a very important factor in early drug discovery, we investigated the effect of APETx4 on hERG currents. APETx4 exerts no inhibitory effect on hERG at a concentration of 1.6 µM. Even at a concentration that is 10 times higher than the IC_50_ for K_V_10.1 (10 µM), the tail current is not significantly inhibited when a prepulse of 40 mV is followed by a pulse to −120 mV. However, since APETx1 displays a voltage-dependent effect on hERG, we investigated the current-voltage relationship of the action of APETx4 on hERG. After addition of 10 µM APETx4, a shift towards more positive potentials was observed. When the non-normalized inhibited current was plotted against the activating prepulse potentials, a maximum inhibition of the inward tail current by 21 ± 5% was observed after a prepulse of −20 mV. At concentrations in the low micromolar range, APETx4 discriminates between K_V_10.1 and hERG.

APETx1, which is highly similar to APETx4 (88% identity), inhibits hERG currents with an IC_50_ value of 34 nM [42]. A 300-fold concentration of APETx4 is not able to inhibit the inward hERG current. APETx1 is also not able to significantly inhibit K_V_10.1, at least not at a concentration of 100 nM [44]. Higher concentration of this toxin might be able to inhibit the K_V_10.1 currents. This would be interesting to explore further in order to fully understand the selectivity of APETx1 for hERG over K_V_10.1 and of APETx4 for K_V_10.1 over hERG. The related toxin APETx3, which only differs from APETx1 by one amino acid and shows an 86% amino acid identity to APETx4, is also not able to inhibit the hERG current at concentrations up to 50 µM [41]. However, this naturally occurring mutant of APETx1 is able to inhibit K_V_10.1 currents by 80% at a concentration of 2 µM (*n* = 2, unpublished data). The only difference between APETx1 and APETx3 is one amino acid substitution from a threonine residue to a proline residue. This single mutation completely changes the selectivity profile of the toxin from a potent hERG inhibitor to a Na_V_ channel modulator. This functional difference can result from the introduction of a structural kink in the peptide by the proline residue [41]. APETx4 contains a threonine residue on the third position, similarly to APETx1. APETx2, a potent ASIC3 inhibitor (IC_50_ ~ 63 nM) [53] and a weaker hERG inhibitor (IC_50_ ~ 1.2 µM) [54], shows no effect on K_V_10.1 at 2 µM (*n* = 3, unpublished data).

### 3.3. The in Vitro Cytotoxic Effect of APETx4 Does not Only Result from Its Effect on K_V_10.1

Similarly to other APETx-like peptides [41,54], APETx4 is not a selective inhibitor. Besides K_V_10.1, APETx4 is also able to inhibit several other K_V_ and Na_V_ channels. However, since natural point mutations in APETx-like peptides can lead to crucial changes in the pharmacological profile of the peptides [41], synthetically produced analogues of APETx4 can result in more potent and more selective K_V_10.1 inhibitors.

The antiproliferative, cytotoxic and proapoptotic effects of APETx4 were evaluated on several cancerous and non-cancerous cell lines. APETx4 was able to induce a dose-dependent effect on all cell lines except on the murine fibroblast cell line NIH-3T3. Even cell lines that presumably do not express K_V_10.1 on its surface were affected. Although the expression levels of K_V_10.1 are not constant during the cell cycle and this makes the interpretation of proliferation and cell viability data more complex [55] we presume that the effect of APETx4 does not only result from its inhibitory activity on K_V_10.1. Interesting to note is that the cytotoxic effects on LNCaP and hTERT RPE-1 cell lines appear very rapidly. The mode of action of cytotoxic peptides can be explained by two nonexclusive mechanisms. The peptides can form new ion channels in the cell membrane and/or they bind to existing ion channels/membrane receptors to modify their activity [56]. Since the cytotoxic effect is not observed in the NIH-3T3 cells or *Xenopus* oocytes, it is unlikely that APETx4 is a cytotoxic channel-forming peptide. We therefore suggest that APETx4 exerts its effect through the binding to unknown membrane proteins. More research is necessary to identify the main targets of APETx4 and to unravel its mode of action.

In conclusion, a novel peptide from the sea anemone *Anthopleura elegantissima* was purified and identified as an APETx peptide. This peptide, named APETx4, is able to inhibit the oncogenic potassium channel K_V_10.1 in a concentration-, voltage- and state-dependent manner. The modulation of K_V_10.1 by APETx4 is reminiscent of the modulation of K_V_11.1 (hERG) by APETx1. However, APETx4 makes a clear distinction between K_V_10.1 and hERG at low micromolar concentrations. APETx4 is not a highly selective K_V_10.1 inhibitor since it is also able to block several K_V_ and Na_V_ channels. Since natural mutants of APETx4 show very different selectivity profiles it could be possible that synthetic analogues show more selectivity for K_V_10.1. Moreover, APETx4 induces a concentration-dependent cytotoxic and proapoptotic effect in various cancerous and in K_V_10.1-expressing cell lines without affecting the proliferation of healthy fibroblast cells. More research is necessary to unravel the mechanism of action of this peptide and to pinpoint its exact binding sites on K_V_10.1 and its other targets. The identification of this novel K_V_10.1 inhibitor can be the first step in the design and synthesis of more potent and selective anticancer compounds.

## 4. Materials and Methods

### 4.1. Purification of APETx4

A crude water-methanol extract of the sea anemone *Anthopleura elegantissima* was fractionated as described previously [37]. These fractions were electrophysiologically screened for activity on K_V_10.1. Active fractions were further purified by reverse-phase high-performance liquid chromatography (RP-HPLC) as described for the purification of APETx3 by Peigneur and colleagues [41].

### 4.2. Biochemical Characterization and Sequence Analysis

The molecular mass of the purified peptide was measured by MALDI-TOF MS (4800 Analyzer, Applied Biosystems) in positive ion reflectron mode and by ESI-FT-ICR MS (SolariX 9.4T, Bruker, Fällanden, Switzerland) analysis in positive ion mode. The peptide sequence was determined by a combination of MALDI- LIFT-TOF/TOF MS/MS (UltrafleXtreme, Bruker) and ESI-FT-ICR MS/MS (CID, SolariX 9.4T, Bruker). For MALDI-LIFT-TOF/TOF experiments, the dried-droplet method was used to deposit the peptide. Less than 1 mg of the peptide has been dissolved into 200 µL of H_2_O/Formic Acid (0.1%), leading to a primary solution (PS) at a concentration below 1 mM. 1 µL of this PS has been deposited together with 1 µL of 2,5-dihydroxibenzoic acid as a matrix (20 mg/mL in 50-50 H_2_O/FA 0.1%-ACN (*v*/*v*) onto a MALDI plate (384 positions, Bruker Daltonics, Bremen, Germany). MS spectra were acquired in reflector mode in the mass range m/z 700–5000 and analyzed by FlexAnalysis 3.4 (Bruker Daltonics software). MS/MS experiments were performed using the LIFT cell for post-acceleration of the metastable fragments. Before MS/MS analysis, the peptide was reduced to remove the disulfide bridges. The reduction was achieved by incubating APETx4 with Tris-(carboxyethyl)phosphine at 200 mM (final concentration) for 50 min at 56 °C. The reduced sample was then purified using a microcolumn ZipTip C18. Exact mass measurements and additional MS/MS experiments have also been conducted using ESI-FT-ICR mass spectrometry. 1 µL of the PS has been diluted with 99 µL of 50-50 H_2_O/FA 0.1%—ACN (*v*/*v*) to reach a concentration of ~10 µM. The acquisition was performed from m/z 100 to 2000. CID experiments were conducted by isolating the [M+4H]^4+^ species detected at *m/z* 1163.75663 and by fragmenting these ions in the collision cell using a collision energy of 35 V. Fragment ions have finally been detected in the ICR cell.

### 4.3. Expression of Voltage-Gated Ion Channels in Xenopus Laevis Oocytes

For the expression of the ion channels (rNa_V_1.4, hNa_V_1.5, mNa_V_1.6, hK_V_1.1, rK_V_1.3, hK_V_1.4, hK_V_1.5, hK_V_2.1, hK_V_10.1a and hK_V_11.1,) in *Xenopus* oocytes, the plasmids were linearized and subsequently transcribed using the T7 or SP6 mMESSAGE-mMACHINE transcription kit (Ambion^®^, Carlsbad, CA, USA).

Stage V-VI *Xenopus laevis* oocytes were isolated by partial ovariectomy. The animals were anesthetized by a 15 min submersion in 0.1% tricaine methane sulfonate (pH 7.0). Isolated oocytes were defolliculated with 2 mg/mL collagenase.

Defolliculated oocytes were injected with 50 nL of cRNA at a concentration of 1 ng/nL using a micro-injector (Drummond Scientific^®^, Broomall, PA, USA). The oocytes were incubated in a solution containing (in mM): NaCl, 96; KCl, 2; CaCl_2_, 1.8; MgCl_2_, 2 and HEPES, 5 (pH 7.4), supplemented with 50 mg/L gentamycin sulfate.

### 4.4. Electrophysiological Recordings

Two-electrode voltage-clamp recordings were performed at room temperature (18–22 °C) using an Geneclamp 500B Voltage and Patch Clamp Amplifier (Axon Instruments, Union City, CA, USA, controlled by a Axon Digidata 1550 Low-Noise Data Acquisition System (Axon Instruments). Whole cell currents from oocytes were recorded 1–4 days after injection. The normal bath solution (ND96) composition was (in mM): NaCl, 96; KCl, 2; CaCl_2_, 1.8; MgCl_2_, 2 and HEPES, 5 (pH 7.4). The high potassium solution (HK) composition was in mM): NaCl, 2; KCl, 96; CaCl_2_, 1.8; MgCl_2_, 2 and HEPES, 5 (pH 7.4). Voltage and current electrodes were filled with 3 M KCl. Resistances of both electrodes were kept between 0.5 and 1.5 MΩ. The elicited K_V_1.x end K_V_2.1 currents were filtered at 500 Hz and sampled at 2 kHz, K_V_10.1 currents were filtered at 1 kHz and sampled at 2 kHz, hERG currents were filtered at 1 kHz and sampled at 10 kHz and the sodium currents were filtered at 2 kHz and sampled at 20 kHz using a four-pole low-pass Bessel filter. Leak subtraction was performed using a -P/4 protocol.

K_V_10.1 currents were evoked by 2 s depolarizing pulses to 0 mV from a holding potential of −90 mV. K_V_11.1 (hERG) peak and tail currents were generated by a 2.5 s prepulse from −90 mV to 40 mV followed by a 2.5 s pulse to −120 mV. K_V_1.x channel currents were evoked by 500 ms depolarizations to 0 mV from a holding potential of −90 mV followed by a 500 ms pulse to −50 mV. K_V_2.1 currents were elicited by 500 ms pulses to +20 mV from a holding potential of −90 mV. Na_V_ currents were evoked by a 100 ms depolarizations to 0 mV from the holding potential.

To investigate the concentration-dependency, the K_V_10.1 current inhibition (%) was measured after addition of 7 varying APETx4 concentrations. The curve was fitted with the logistic dose-response equation, *y* = $\frac{A_{1}-A_{2}}{1+\;{\left({\mathrm{IC}}_{50}/\left[\mathrm{toxin}\right]\right)}^{n_{H}}}$ + *A*_2_ where *y* represents the percentage of current inhibition, *A*_1_ the initial inhibition at the lowest toxin concentration (0%), *A*_2_ the final inhibition at the highest toxin concentration, IC_50_ the half maximal inhibitory toxin concentration and *^n^*_H_ the Hill coefficient.

The voltage-dependent activation of the K_V_10.1 channels was investigated by 1000 ms activating steps from the holding potential −90 mV to 65 mV with 5 mV increments. The normalized current amplitudes were plotted against the corresponding pulse potentials and fitted with the Boltzmann equation, *y* = $\frac{A_{1}-A_{2}}{1+{\;e}^{\left(V-V_{1/2}\right)/k}}$ + *A*_2_, where y represents the normalized current (I/I_max_), *A*_1_ is the initial *y*-value(-∞) and *A*_2_ is the final *y*-value, I_max_ is the maximal current, *V* is the test voltage, *V*_1/2_ is the half-maximal voltage and *k* is the slope factor.

The voltage-dependent inactivation of K_V_10.1 channels was studied by 1.5 s prepulses from the holding potential ranging from −140 mV to 30 mV with 10 mV steps. These pulses were followed by a 0.5 s test pulse to 30 mV [17]. The normalized peak current amplitudes (I_2_/I_2,max_) measured during the test pulse and were plotted against the prepulses and fitted with the Boltzmann equation.

The peak and tail current of hERG were measured at different voltages by 1500 ms activating pulses from −60 mV to 55 mV in 5 mV steps from the holding potential of −90 mV, followed by a 750 ms step to −120 mV.

### 4.5. Live Cell Imaging

#### 4.5.1. Cell Cultures

Cell lines SH-SY5Y (ACC 209), LNCaP (ACC 256) and NIH-3T3 (ACC 59) were purchased from DSMZ (Germany). MDA-MB-435S (HTB 129) and hTERT RPE-1 (CRL 4000) were obtained from ATCC (USA). Cell lines were cultured in their recommended media supplemented with 10% or 15% FCS (PAA laboratories, Cölbe, Germany) at 37 °C in humidified 5% CO_2_ atmosphere. An overview of the used cell lines is given in Table 2. All media were purchased from ThermoFisher Scientific (Waltham, MA, USA).

#### 4.5.2. Proliferation, Cytotoxicity and Apoptosis Assays

Cell proliferation, cytotoxicity and apoptosis were assessed in a 96-well microtiter plate by live-cell imaging using an IncuCyte Zoom System (Essen BioScience, Welwyn Garden City, UK). Cell proliferation was monitored in terms of cell confluency (%). Cell cytotoxicity was assessed by the CellTox Green Dye assay (Promega, Madison, WI, USA). The IncuCyte Caspase-3/7 apoptosis assay (Essen BioScience, UK) was used to evaluate the effect of APETx4 on the apoptotic pathway.

### 4.6. Data Analysis and Molecular Modelling

All electrophysiological data are presented as means ± S.E.M of *n* ≥ 3 independent experiments unless otherwise indicated. All data were acquired using pClamp Clampex 10.4 Molecular Devices, Downingtown, PA, USA) and analyzed using pClamp Clampfit 10.4 (Molecular Devices) and OriginPro 8 (Originlab, Northampton, MA, USA) or GraphPad Prism 6 software (GraphPad Software, Inc., San Diego, CA, USA). Paired student’s *t*-tests were performed to compare 2 sample means (*p* < 0.05).

Live-cell imaging data were collected from the IncuCyte Zoom software and analyzed using GraphPad Prism 6 software. Proliferation was measured as Phase Object Confluence (%), cytotoxicity and apoptosis were measured as Green Object Count (1/mm²). Data points were collected every hour. In order to improve the clarity of the graphs, only the data points for every 5 h are shown. All data are represented as mean ± S.E.M of *n* = 4 different wells.

Homology modeling was performed using Chimera 1.10.1 [57] enhanced with the Modeller 9.14 software [58].

Clustal Omega multiple sequence alignments were performed using CLC Main Workbench (QIAGEN, Aarhus, Denmark) or EMBL-EBI [59]. The following UNIPROT entry identifiers were used for Figure 2; P61541, B3EWF9, P86461, P61542, G0W2H8, P86470, G0W2H8, P86464, P86462, P84919 and Figure 8; P61541, B3EWF9, P61542, P60590, P60980, P83480, P56854, P56852.

## Figures and Tables

**Table 1:** Cell lines used for proliferation, cytotoxicity and apoptosis assays.

Cell Line	Description	K_V_10.1 Expression Level	Ref.
SH-SY5Y	Human neuroblastoma cell line	High	[20]
LNCaP	Human prostate cancer cell line	Undetectable	[20]
NIH-3T3	Mouse embryonic fibroblast cell line	Undetectable	[14]
MDA-MB-435S	Human melanoma cell line	Moderate	[20]
hTERT RPE-1	Human epithelial cell line	Moderate	[20]

**Table 2:** Cell lines used for proliferation, cytotoxicity and apoptosis assays.

Cell Line	Description	Medium
SH-SY5Y	Human neuroblastoma cell line	RPMI + 15% FCS
LNCAP	Human prostate cancer cell line	RPMI + 15% FCS
NIH-3T3	Mouse embryonic fibroblast cell line	DMEM + 10% FCS
MDA-MB-435S	Human melanoma cell line	RPMI + 10% FCS
hTERT RPE-1	Human epithelial cell line	DMEM:F12 + 10% FCS
		+ 10 µg/mL Hygromycin B

## Supplementary Materials

The following are available online at www.mdpi.com/1660-3397/15/9/287/s1, Figure S1. Determination of the monoisotopic molecular mass of APETx4, Figure S2. ESI-FT-ICR spectra of oxidized and reduced APETx4, Figure S3. Homology modeling of APETx4, Figure S4. Selectivity screening on a panel of Na_V_ and K_V_ channels, Figure S5. Investigation of the antiproliferative and cytotoxic effects of APETx4 on various cell lines. Figure S6. Cell images of various cell lines during apoptosis experiments.
