# Supplementary material for: APETx4, a Novel Sea Anemone Toxin and a Modulator of the Cancer-Relevant Potassium Channel KV10.1
Source: Mar Drugs. 2017 Sep 13;15(9):287. doi: 10.3390/md15090287 (PMC5618426; doi:10.3390/md15090287)
Supplement: Supplementary file 1 [file marinedrugs-15-00287-s001.zip › Figure S6.pdf]

SH-SY5Y - Caspase assay

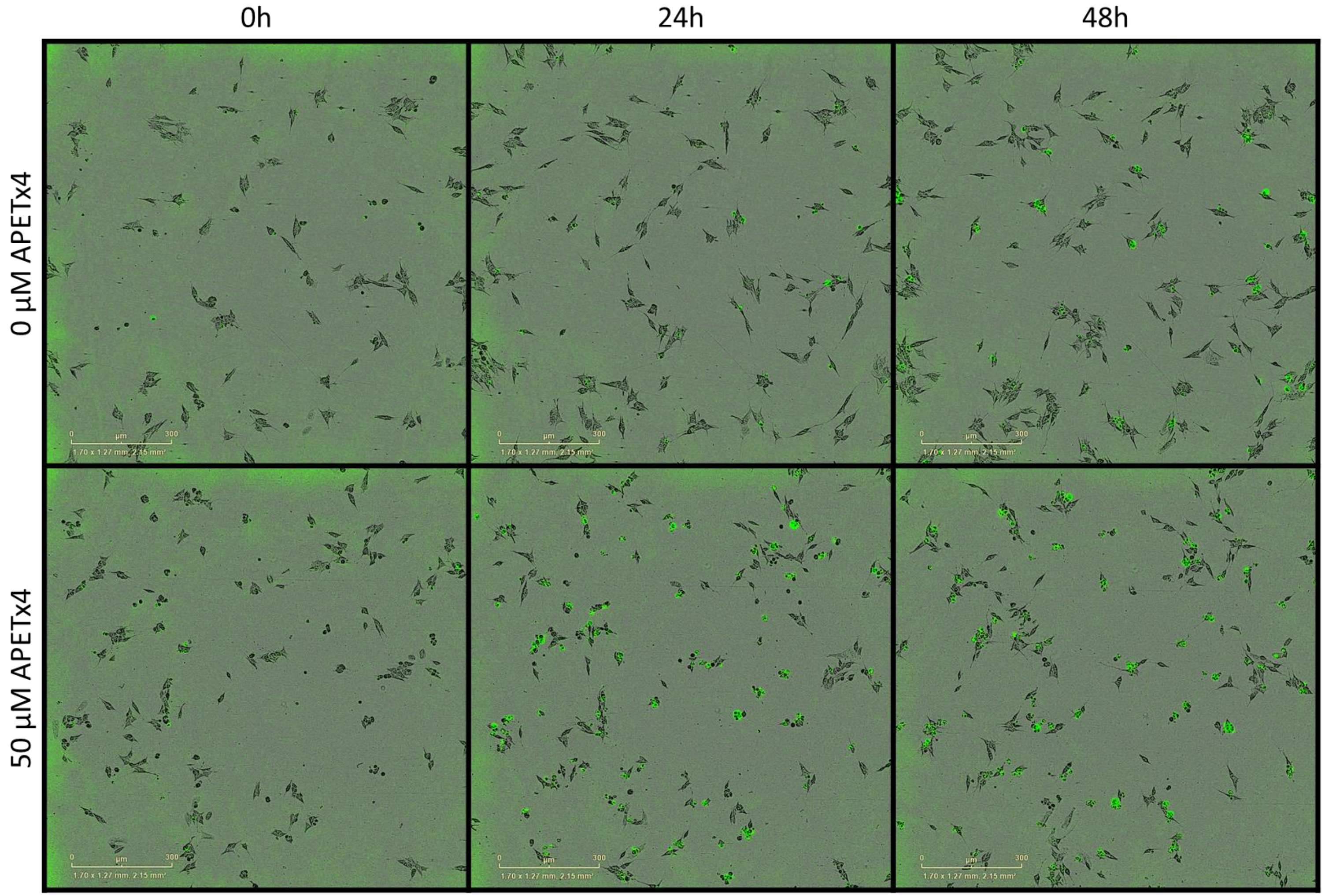

MDA-MB-435S - Caspase Assay

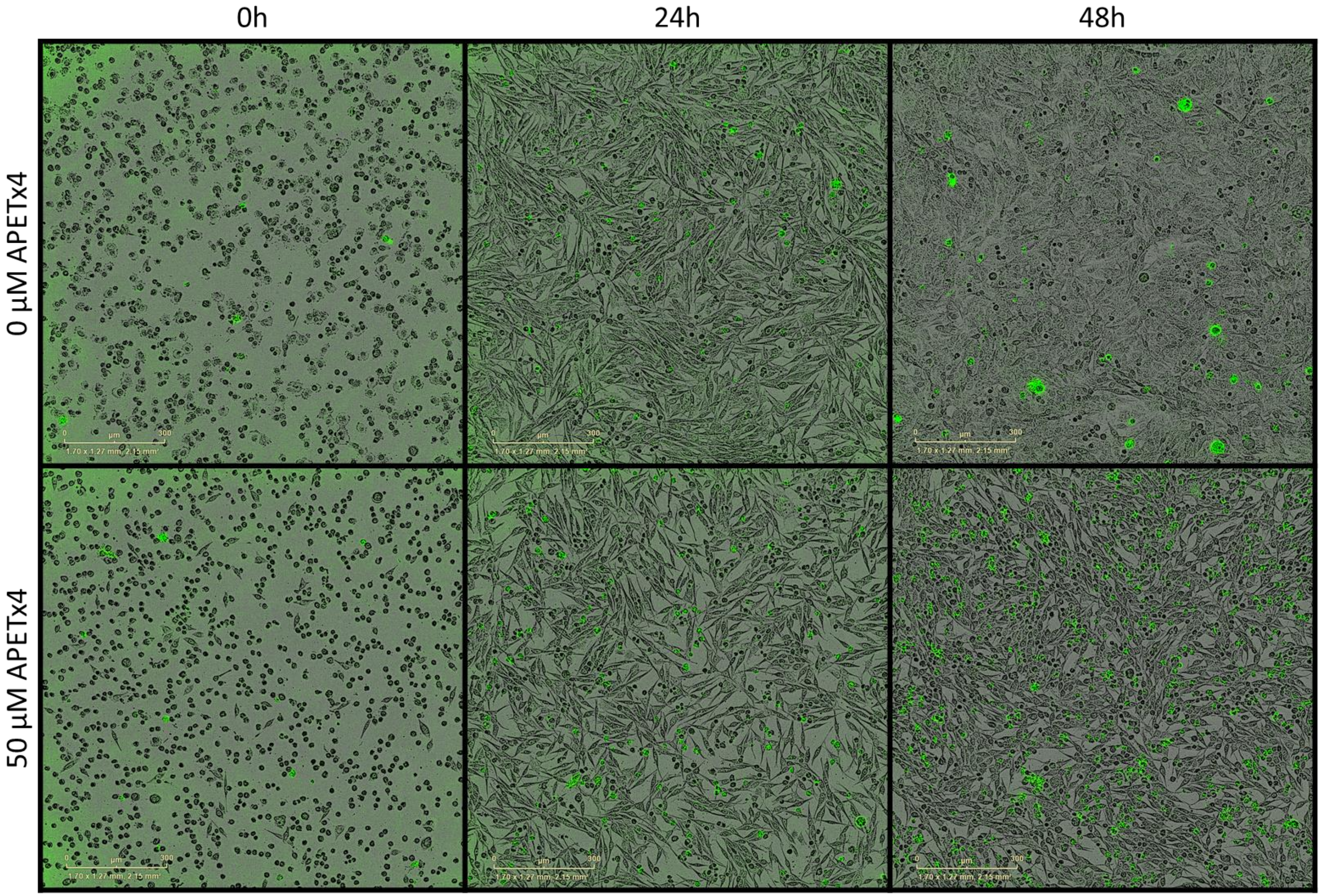

LNCAP - Caspase Assay

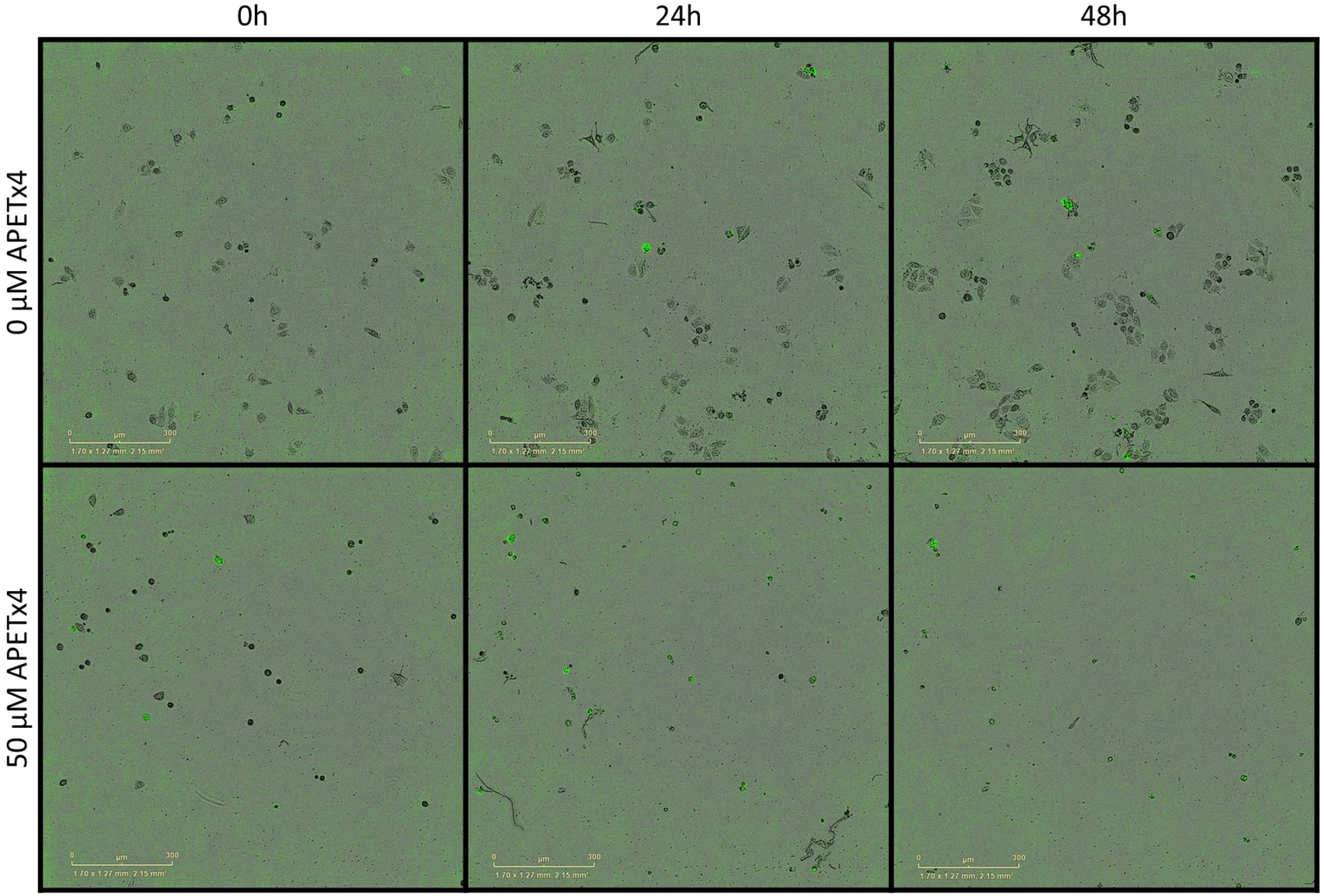

hTERT RPE-1 - Caspase Assay

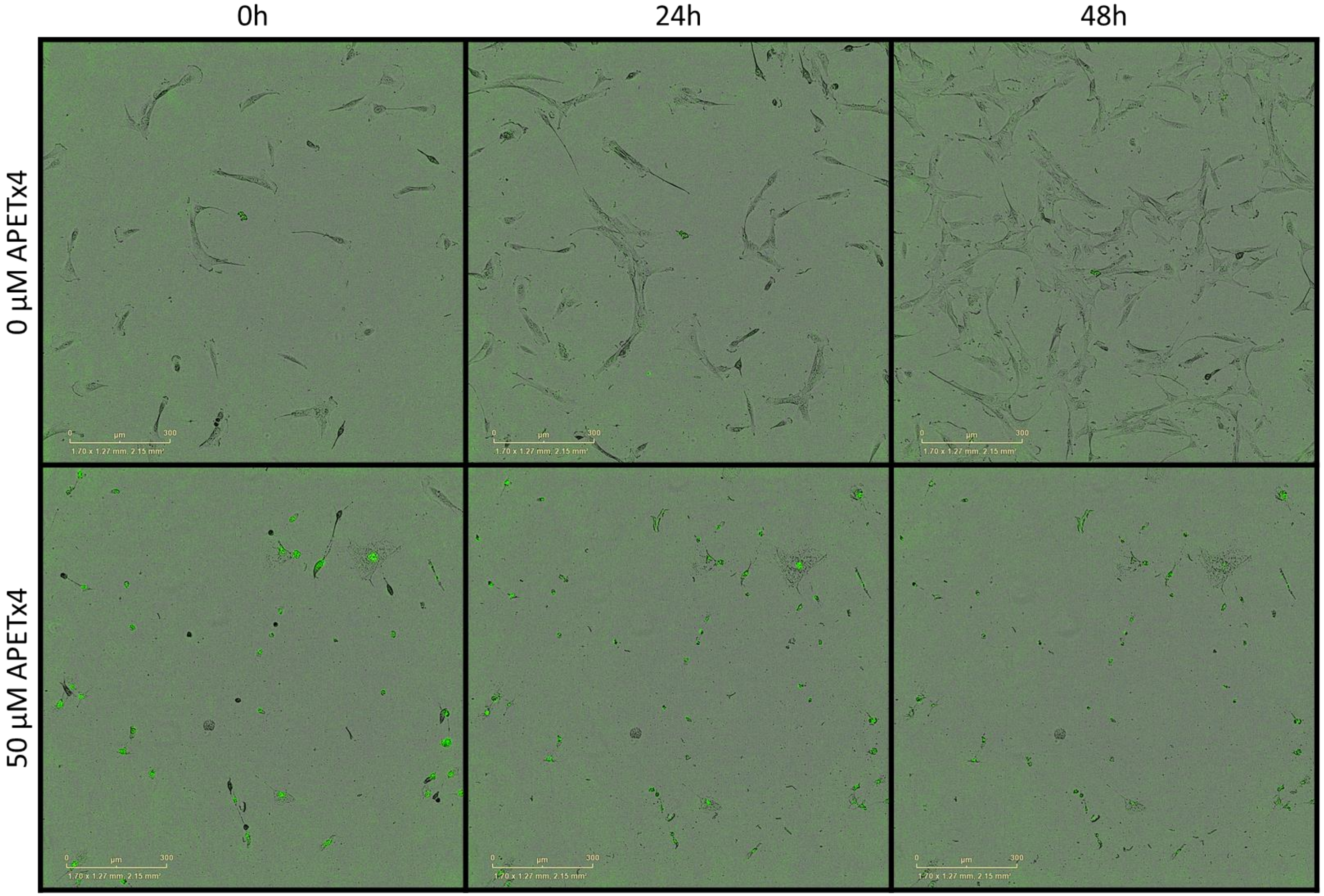

NIH-3T3 - Caspase assay

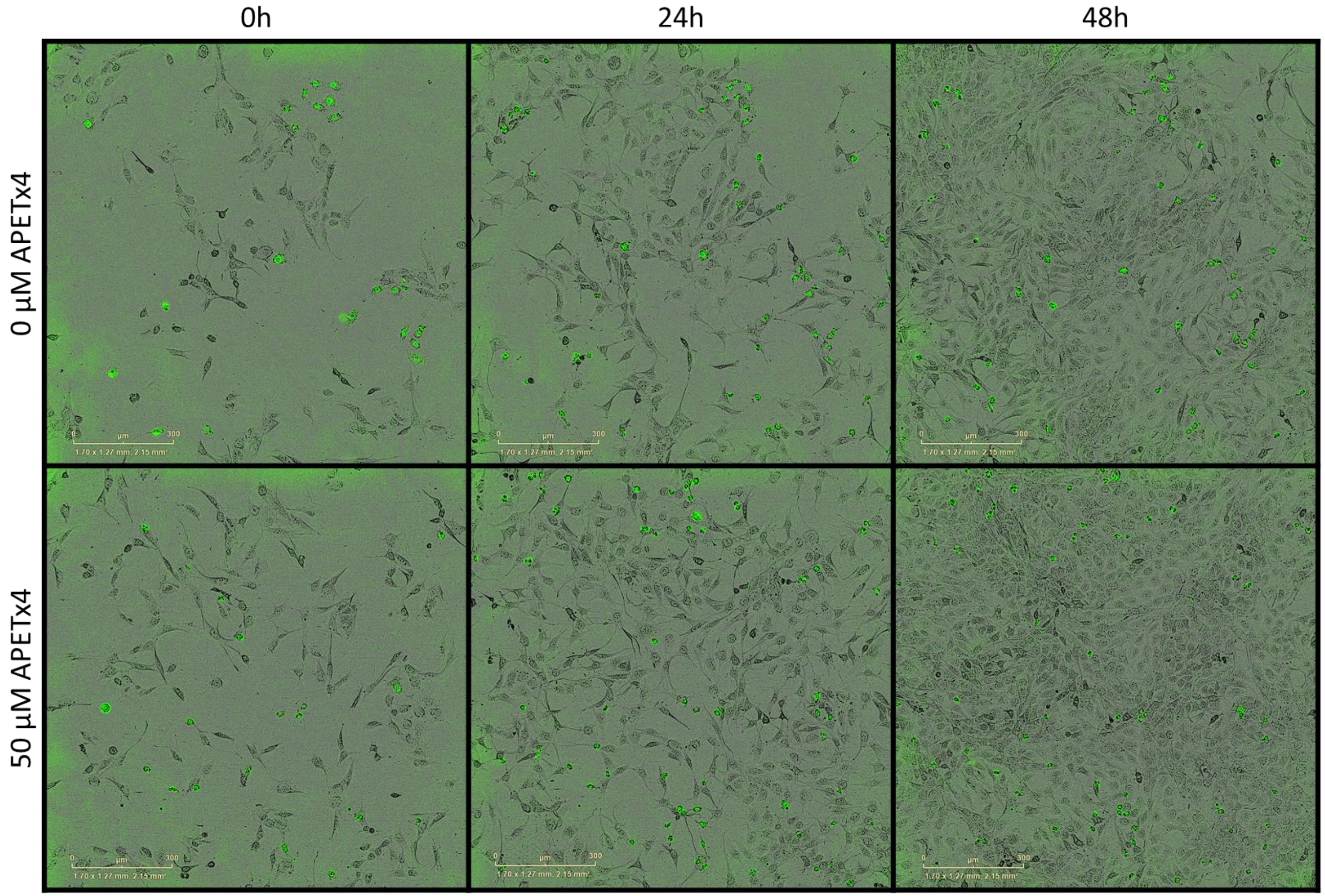

Figure S6. Cell images of various cell lines during apoptosis experiments. Images were taken 0h, 24h and 48 h after addition of 0 or 50 µM APETX4.
