# Supplementary material for: APETx4, a Novel Sea Anemone Toxin and a Modulator of the Cancer-Relevant Potassium Channel KV10.1
Source: Mar Drugs. 2017 Sep 13;15(9):287. doi: 10.3390/md15090287 (PMC5618426; doi:10.3390/md15090287)
Supplement: Supplementary file 1 [file marinedrugs-15-00287-s001.zip › Figure S1.pdf]

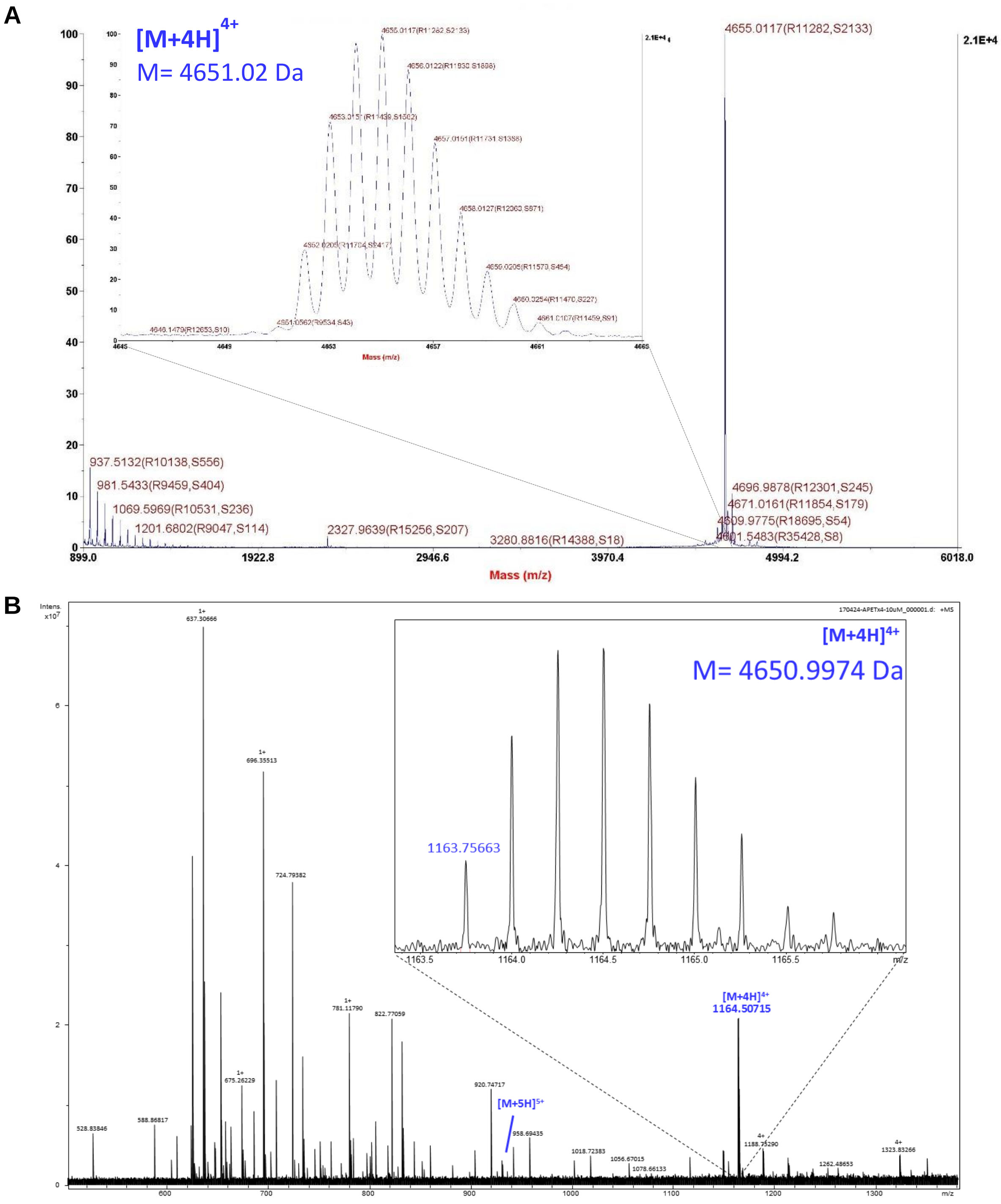

Figure S1. Determination of the monoisotopic molecular mass of APETx4 by (**a**) MALDI TOF MS (**b**) ESI-FT-ICR MS. FT-ICRlead to a peptide mass of 4650.9974 Da, which perfectly fits (1.3ppm) with the proposed APETx-4 (M=4650.9914 Da).
