# Supplementary material for: APETx4, a Novel Sea Anemone Toxin and a Modulator of the Cancer-Relevant Potassium Channel KV10.1
Source: Mar Drugs. 2017 Sep 13;15(9):287. doi: 10.3390/md15090287 (PMC5618426; doi:10.3390/md15090287)
Supplement: Supplementary file 1 [file marinedrugs-15-00287-s001.zip › Figure S2.pdf]

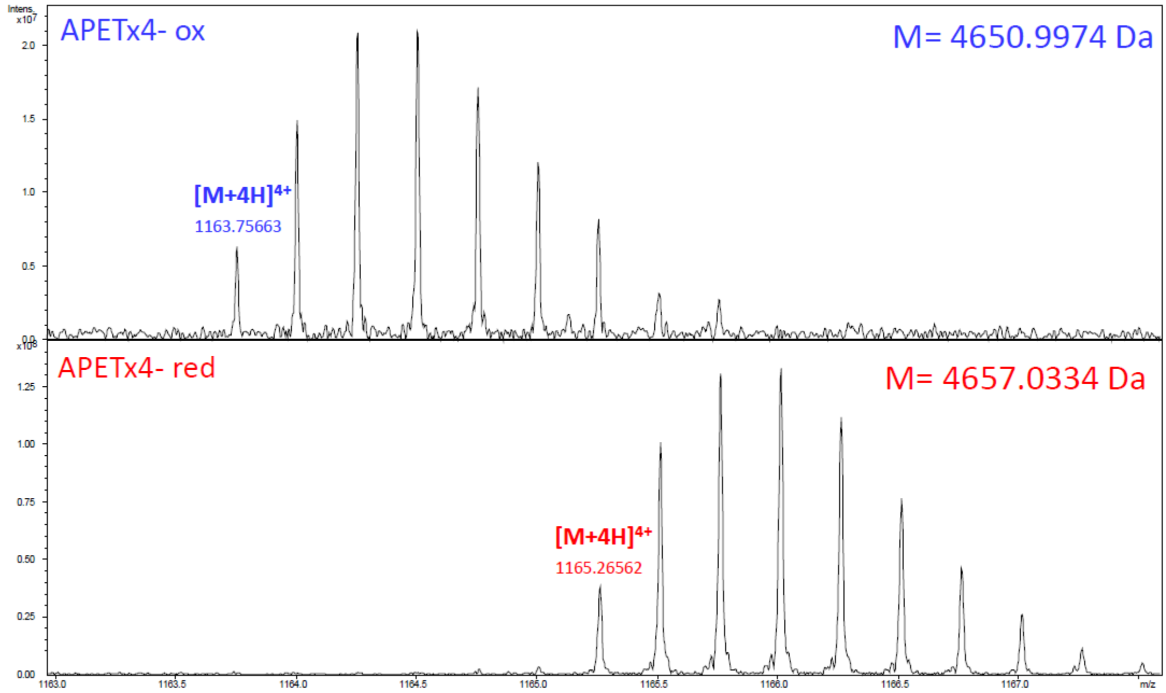

Figure S2. ESI-FT-ICR spectra of oxidized (*Top*) and reduced (*Bottom*) APETx4, focusing on the $[M+4H]^{4+}$ species. Each reduced disulfide bond leads to the molecular weight increase by 2.016 Da. The difference between the masses of the two species charged with four proton clearly indicates that APETx4 has three disulfide bonds (Number of S-S = (1165.26562-1163.75663)*4/2.016 = 3).
