# Supplementary material for: APETx4, a Novel Sea Anemone Toxin and a Modulator of the Cancer-Relevant Potassium Channel KV10.1
Source: Mar Drugs. 2017 Sep 13;15(9):287. doi: 10.3390/md15090287 (PMC5618426; doi:10.3390/md15090287)
Supplement: Supplementary file 1 [file marinedrugs-15-00287-s001.zip › Figure S3.pdf]

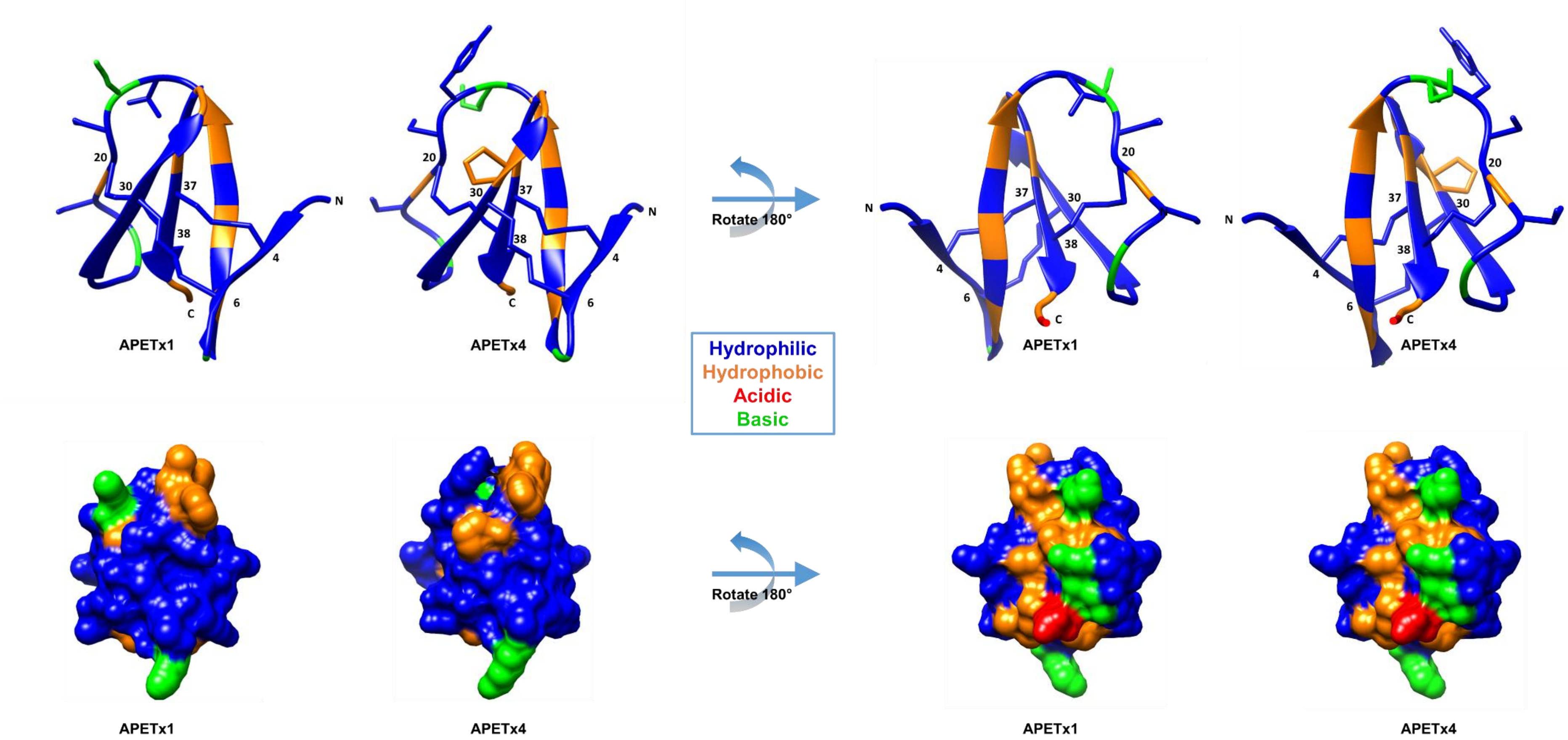

Figure S3. Homology modelling of APETx4. The amino acid sequence of APETx4 was modelled on an averaged structured obtained from the solution NMR structure of APETx1 (PDB ID: 1WQK) by using Modeller and Chimera. The amino acid residues are colored based on their properties. Hydrophilic residues are shown in blue, hydrophobic residues in orange, acidic residues in red and basic residues in green. The 5 amino acid residues that differ between APETx1 and APETx4 are displayed as sticks. The C- and N-terminal residues and the cysteine residues (4, 6, 20, 30, 37 and 38) are indicated on the upper panel. In the lower panel the solvent-excluded molecular surfaces of the peptides are shown.
