# Supplementary material for: APETx4, a Novel Sea Anemone Toxin and a Modulator of the Cancer-Relevant Potassium Channel KV10.1
Source: Mar Drugs. 2017 Sep 13;15(9):287. doi: 10.3390/md15090287 (PMC5618426; doi:10.3390/md15090287)
Supplement: Supplementary file 1 [file marinedrugs-15-00287-s001.zip › Figure S4.pdf]

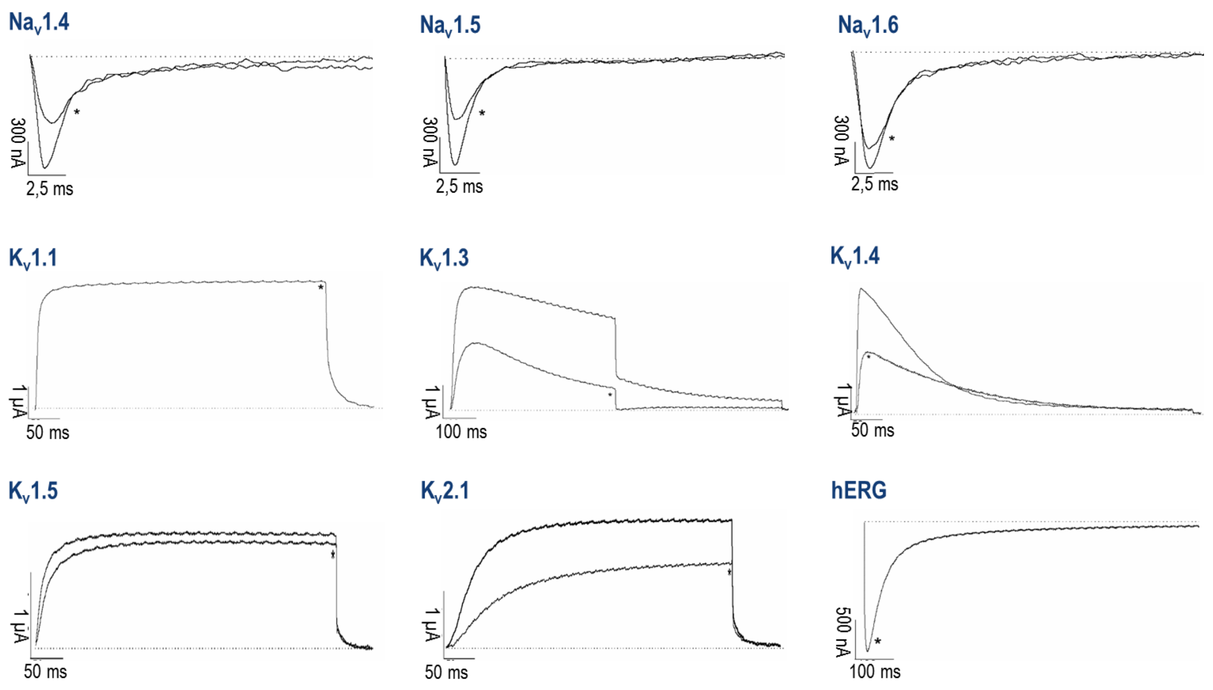

Figure S4. Selectivity screening on a panel of Nav and Kv channels. Representative whole-cell current traces in control and toxin conditions (*) are shown. The traces in toxin condition are representative steady-state traces after application of 1.6 μM toxin.
