# Supplementary material for: APETx4, a Novel Sea Anemone Toxin and a Modulator of the Cancer-Relevant Potassium Channel KV10.1
Source: Mar Drugs. 2017 Sep 13;15(9):287. doi: 10.3390/md15090287 (PMC5618426; doi:10.3390/md15090287)
Supplement: Supplementary file 1 [file marinedrugs-15-00287-s001.zip › Figure S5.pdf]

A

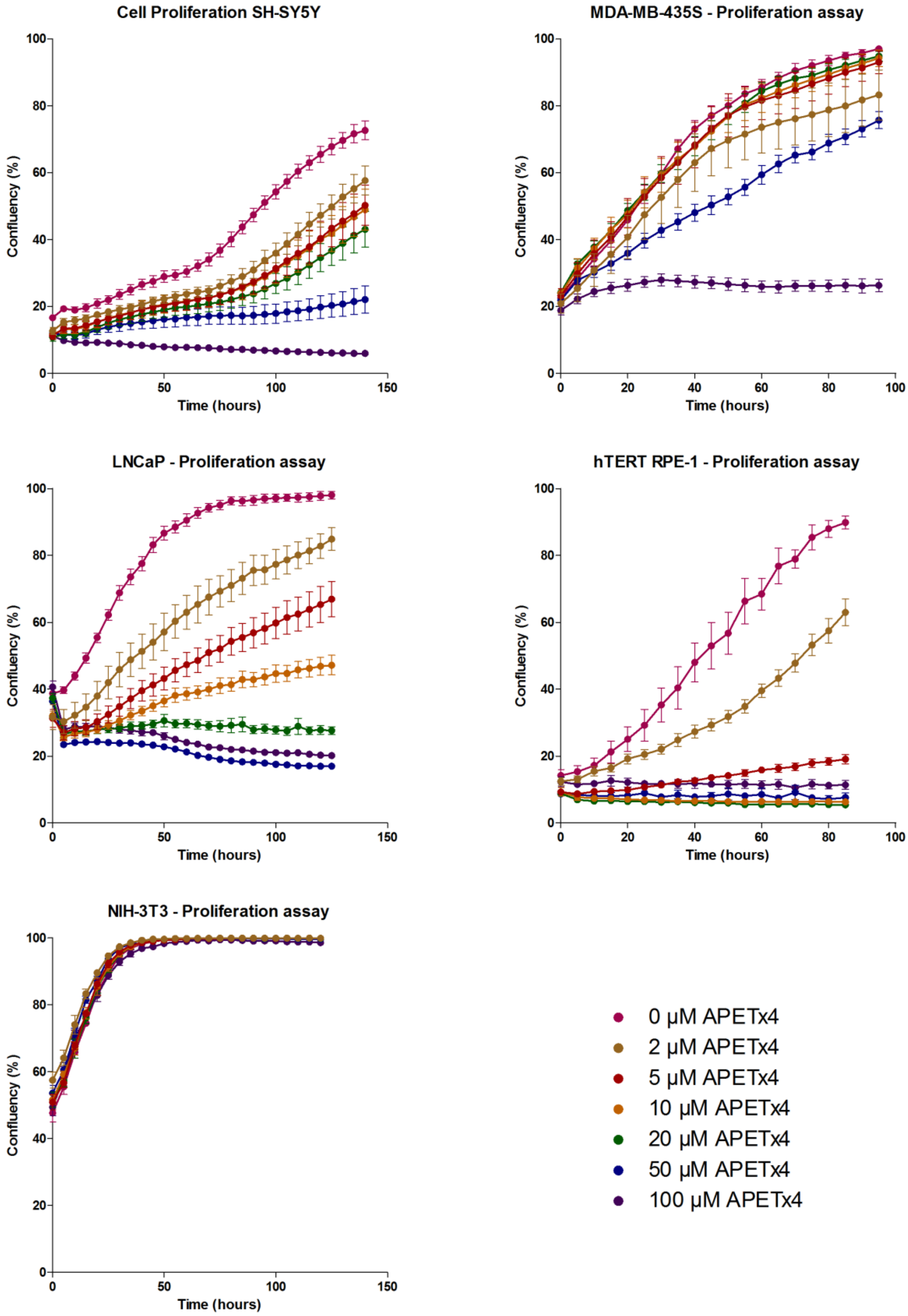
Cell Proliferation SH-SY5Y
MDA-MB-435S - Proliferation assay
LNCaP - Proliferation assay
hTERT RPE-1 - Proliferation assay
NIH-3T3 - Proliferation assay
Confluency (%)
Time (hours)
0 µM APETx4
2 µM APETx4
5 µM APETx4
10 µM APETx4
20 µM APETx4
50 µM APETx4
100 µM APETx4

B

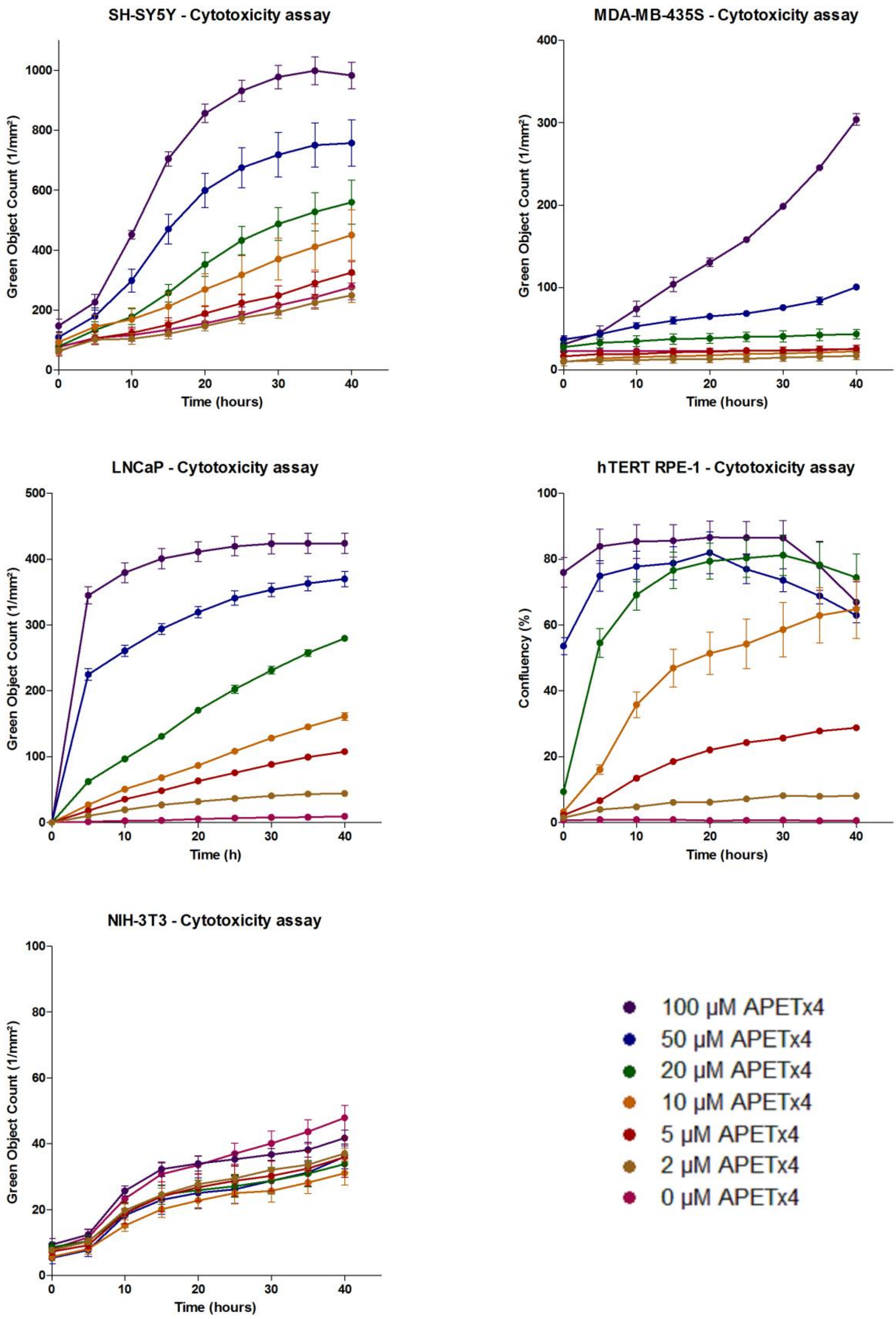

Figure S5. Investigation of the antiproliferative (A) and cytotoxic (B) effects of APETx4 on various cell lines.
